# Atypical Multidrug-Resistant Salmonella paratyphi B Infection in a Patient with Uncontrolled Diabetes Mellitus: A Case Report

**DOI:** 10.7759/cureus.66000

**Published:** 2024-08-02

**Authors:** Nandhini Ravella Venkatasubramanyam, Lavanya Ramanan, Neelusree Prabhakaran, Manivannan Nandhagopal

**Affiliations:** 1 Department of Microbiology, Saveetha Medical College and Hospital, Saveetha Institute of Medical and Technical Science, Chennai, IND

**Keywords:** wound infection, salmonella paratyphi b, multidrug resistance salmonella, foot ulcer, atypical salmonella

## Abstract

Atypical *Salmonella* infection usually presents with unusual symptoms in addition to gastroenteritis. Such atypical presentations can pose a challenge for diagnosis and treatment as they may be misdiagnosed, leading to delayed care and potential complications. Here we report an unusual case of *Salmonella* spp. isolated from a wound swab. A 57-year-old male patient with a history of uncontrolled type 2 diabetes presented to the general surgery department with a 25-day history of swelling, ulceration, and purulent discharge on his right foot. A wound swab was collected for culture and sensitivity. Gram staining showed occasional pus cells and a few gram-negative bacilli. Culture was done, and the organism was identified as *Salmonella *Paratyphi B with the help of other biochemicals. The isolate showed susceptibility to chloramphenicol and cotrimoxazole and resistance to other panels of antibiotics. Routine blood and urine analysis of the patient showed normal findings. Wound dressing was done on an alternative day, followed by administration of antibiotics. The patient was advised to follow up after two weeks. The clinical outcome in the above patient was satisfactory with appropriate antibiotics. We present a case of atypical typhoidal *Salmonella *as a rare cause of wound infection and not a major threat if diagnosed and treated accordingly.

## Introduction

Enteric fever is a systemic infectious disease caused by *Salmonella *species that affected around 16 million people globally in the 1990s [1]. The rise in multi-drug-resistant (MDR) strains poses a significant risk to public health [2, 3]. In the United States, the Centers for Disease Control and Prevention estimated that around 21.6 million typhoid cases are reported annually, with the yearly incidence ranging from 100 to 1,000 cases per 100,000 people [3, 4]. Globally, enteric fever causes an estimated 600,000 deaths annually. In India, the common types of *Salmonella* responsible for enteric fever are *Salmonella *Typhi, *Salmonella *Paratyphi A, and *Salmonella *Paratyphi B.

The clinical features of typhoid include step ladder fever, headache, abdominal pain, tiredness, weight loss, cough, and diarrhea. The bacteria invade the intestinal mucosa mainly through microfold (M) cells [5]. The invasive pathogens result in the trigger of a rapid inflammatory or diarrheal response. The mucosal inflammatory response is a key clinical feature of salmonellosis. Typhoid fever is generally difficult to differentiate from other fevers like malaria clinically. A clinician who understands various febrile diseases and their distinct clinical patterns can play a crucial role in identifying the underlying cause of illness [6]. Diabetes mellitus, when uncontrolled, can impair the immune response, making it difficult to clear the infections, contributing to the atypical presentation/focal salmonellosis leading to the isolation of *Salmonella *from diabetic foot ulcers. Management of focal infections includes prompt initiation of antibiotic therapy based on their susceptibility profile and resistance. Understanding antimicrobial resistance (AMR) in typhoidal *Salmonella *is crucial for effective clinical management. Recent literature has shown only a few instances where *Salmonella *species were isolated from diabetic ulcers. The common organism isolated from diabetic ulcers was *Salmonella *Typhi [7].

In an atypical *Salmonella *infection, the patient usually presents with unusual symptoms in addition to gastroenteritis. Such atypical presentations can pose a challenge for diagnosis and treatment as they may be overlooked or misdiagnosed, leading to delayed care and potential complications. Ideal management would include targeted antibiotic therapy based on their susceptibility profile to *Salmonella *infection and alternative wound dressings. Early recognition and management of atypical cases can prevent further transmission and reduce the risk of severe illness or long-term complications. In this study, we reported a case of a patient with atypical MDR *Salmonella* Paratyphi B infection and uncontrolled diabetes.

## Case presentation

**Figure 1:**
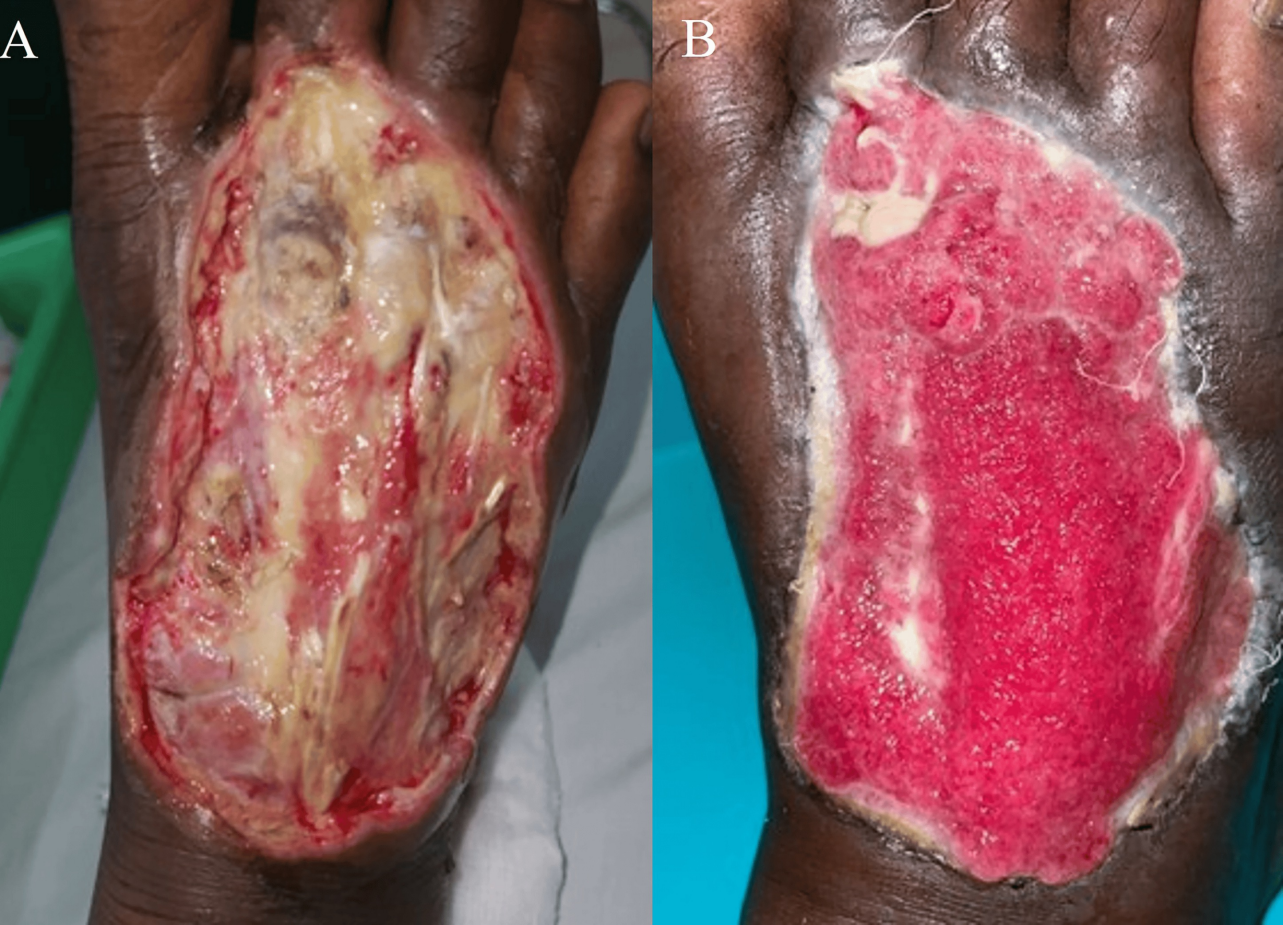
Diabetic foot ulcer of the patient pre-treatment and post treatment A. Image shows the patient's left foot before treatment. The ulcer appears extensive with a significant amount of necrotic tissue and purulent discharge. The wound is surrounded by inflamed, reddened skin, indicating an active infection; B. Image depicts the same foot after treatment. The ulcer significantly improved after two weeks, showing a clean, granulating wound bed with minimal necrotic tissue. The surrounding skin appears less inflamed, indicating a reduction in infection and a positive response to the treatment.

A 57-year-old male with uncontrolled diabetes visited the general surgery department. He reported a high-grade fever, accompanied by fatigue and muscle pain, and a 25-day history of swelling and an ulcer on his right foot with purulent discharge, as shown in Figure 1.

Upon admission, the patient was conscious, alert, and in stable condition. His examination revealed a body temperature of 39 °C, blood pressure of 100/70 mmHg, a heart rate of 92 beats per minute, a respiratory rate of 20 breaths per minute, and an oxygen saturation level of 96% on room air. Upon physical examination, he appeared pale with no signs of jaundice, or dry mucous membranes, and no swelling, lymphadenopathy, or skin rash were detected. The baseline investigations were done and are documented in Table 1.

**Table 1:** Laboratory investigations of the patient CRP: C-reactive protein; ESR: erythrocyte sedimentation rate

Investigations	Reference range	Patient's values
Hemoglobin	13.2–16.6 gm/dl	13.8 gm/dl
Total leucocyte count	4,000–10,000 /µL	12500 / µL
Platelet count	1,50,000–4,10,000 /µL	2,00,000/µL
Serum urea	17–49 mg/dL	18 mg/dL
Serum creatinine	0.6–1.35 mg/dL	0.9 mg/dL
CRP	<0.5 mg/dl	130 mg/dl
ESR	0-20 mm/hour	60 mm/hour

**Figure 2:**
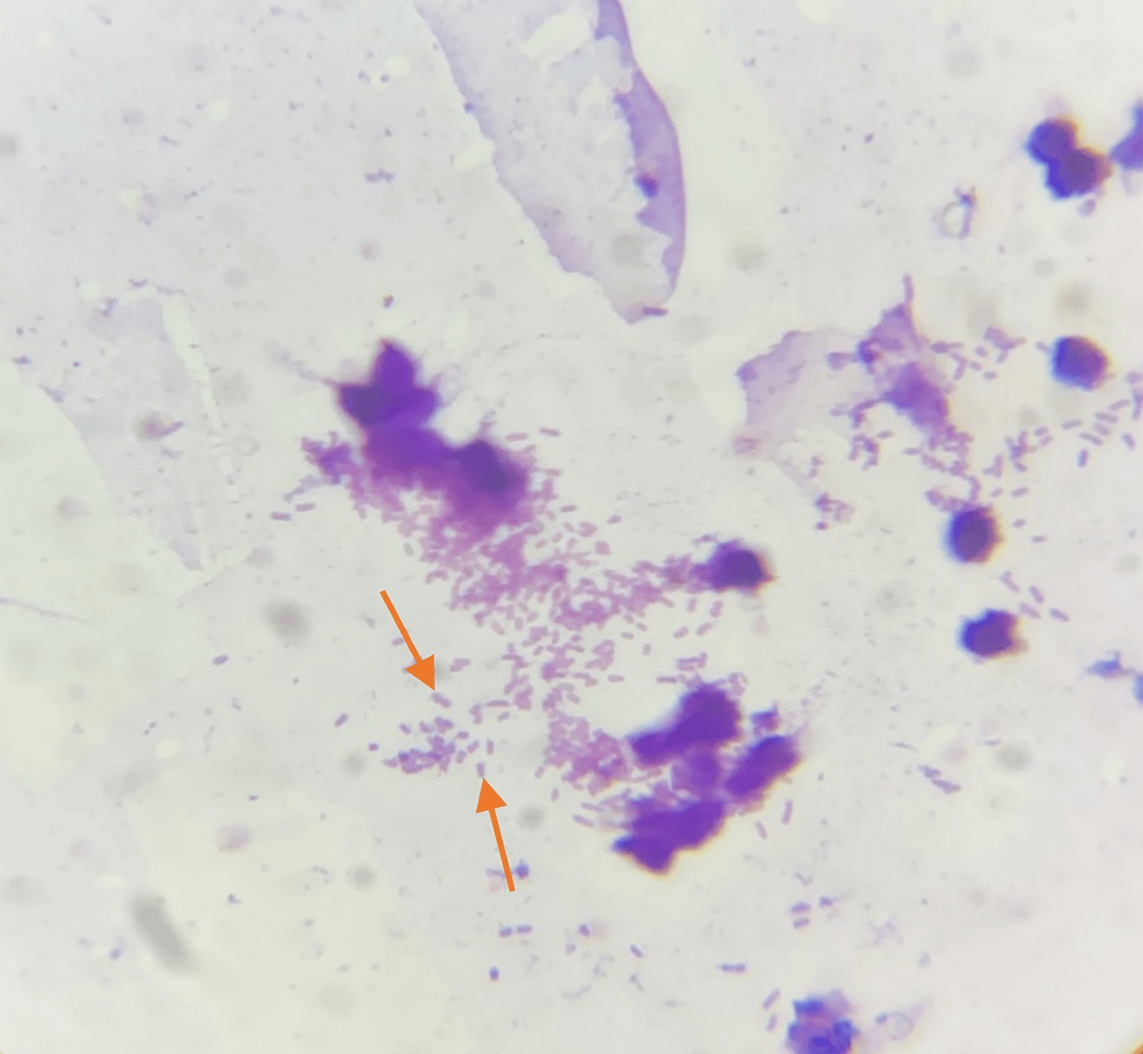
Gram staining shows occasional pus cells and a few gram-negative bacilli

A wound swab was obtained from a diabetic foot ulcer. Initially, the wound area was cleansed with sterile saline to eliminate debris and excess exudate. Using a sterile cotton swab, the swab was gently rolled over the wound bed to collect the sample, which was then placed in a sterile container and sent for culture and sensitivity testing. The specimen was subjected to Gram staining, culture media inoculation, biochemical identification, and susceptibility testing. A provisional diagnosis of a right diabetic foot ulcer was made, and the patient was empirically started on intravenous meropenem 1 g twice a day. Gram staining revealed occasional pus cells and a few gram-negative bacilli (Figure 2). 

**Figure 3:**
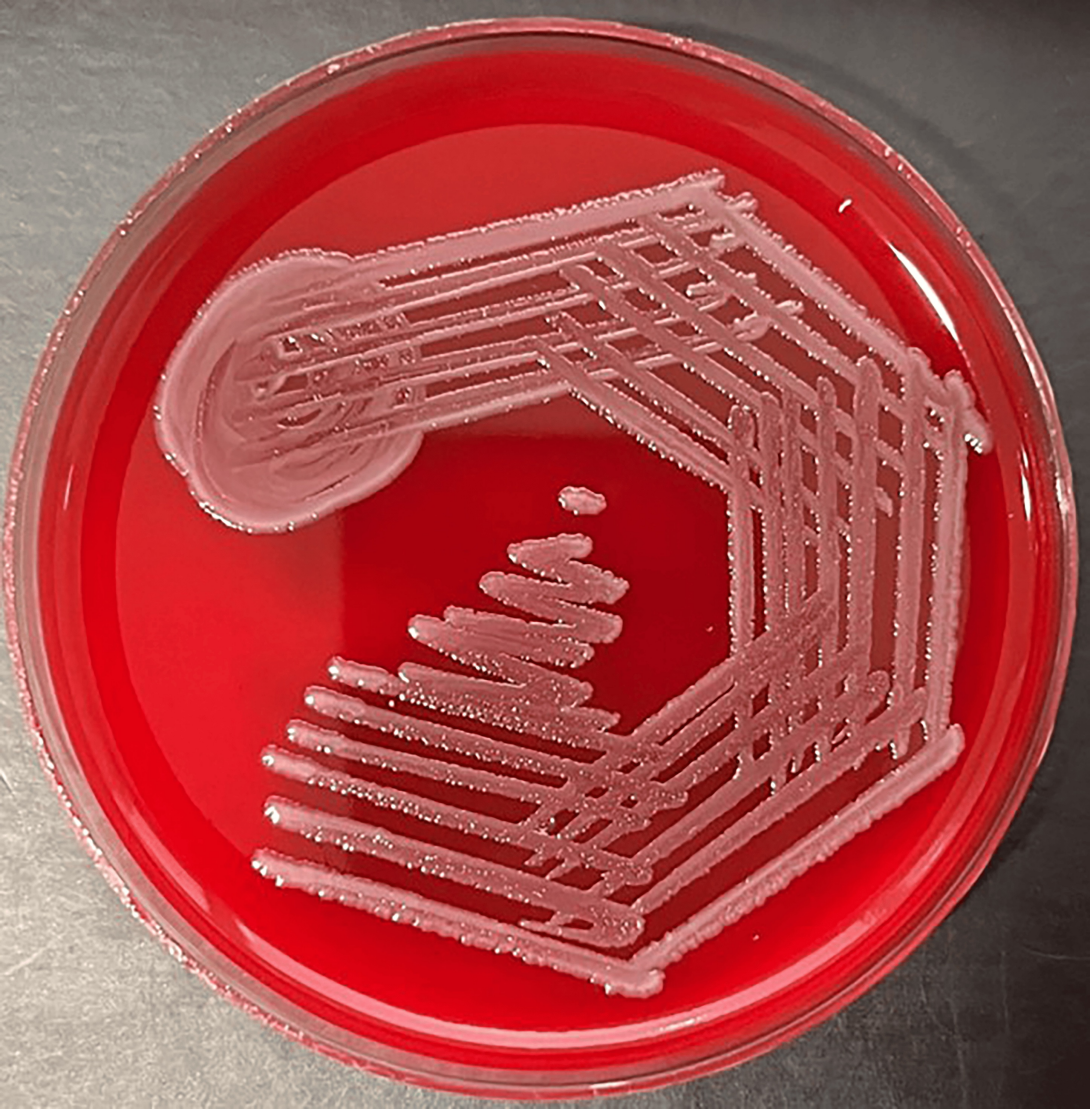
Blood agar shows non-hemolytic grey colonies

**Figure 4:**
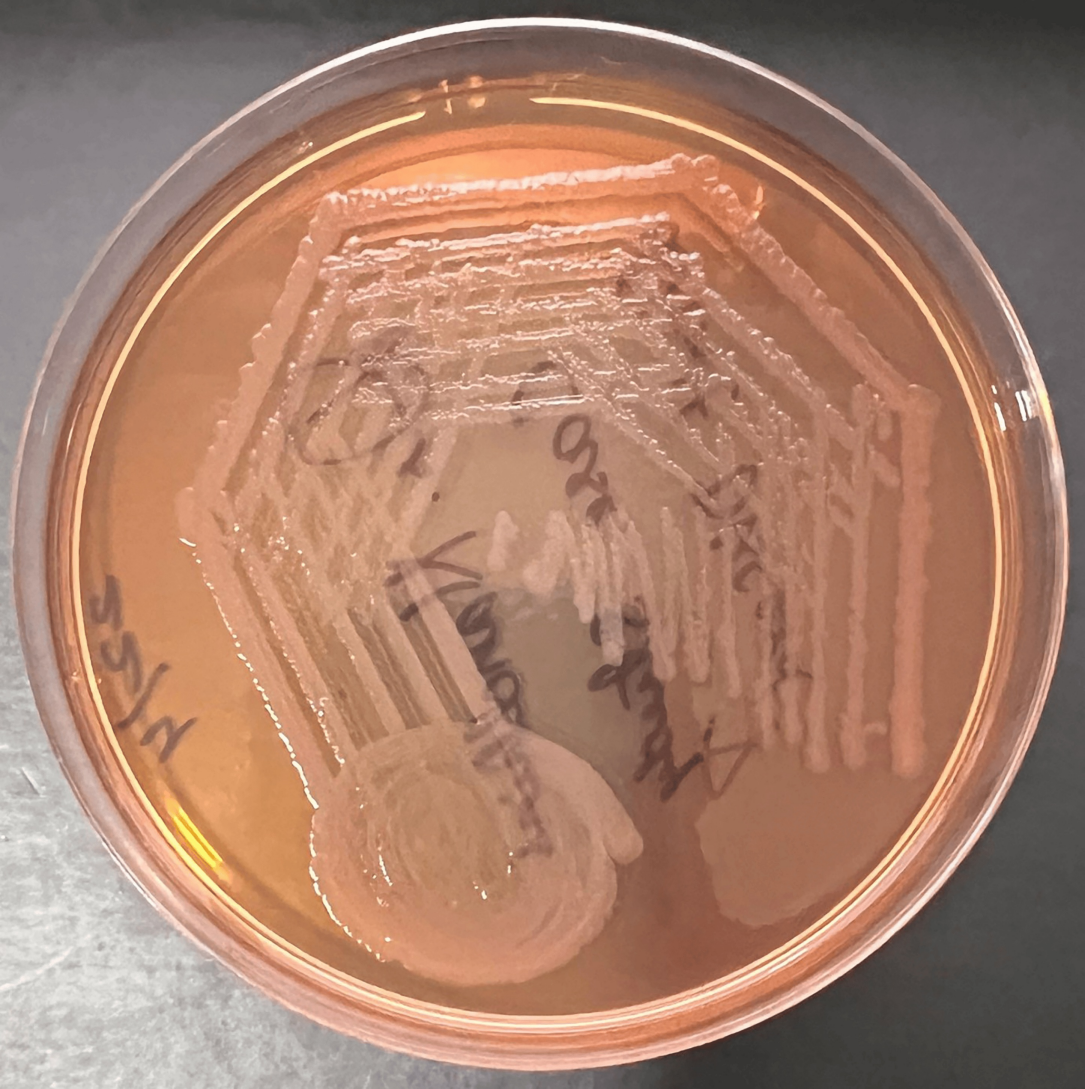
MacConkey agar shows non-lactose fermenting colonies

Five percent sheep blood agar showed non-hemolytic gray colonies (Figure 3), while MacConkey agar showed non-lactose fermenting colonies (Figure 4).

**Figure 5:**
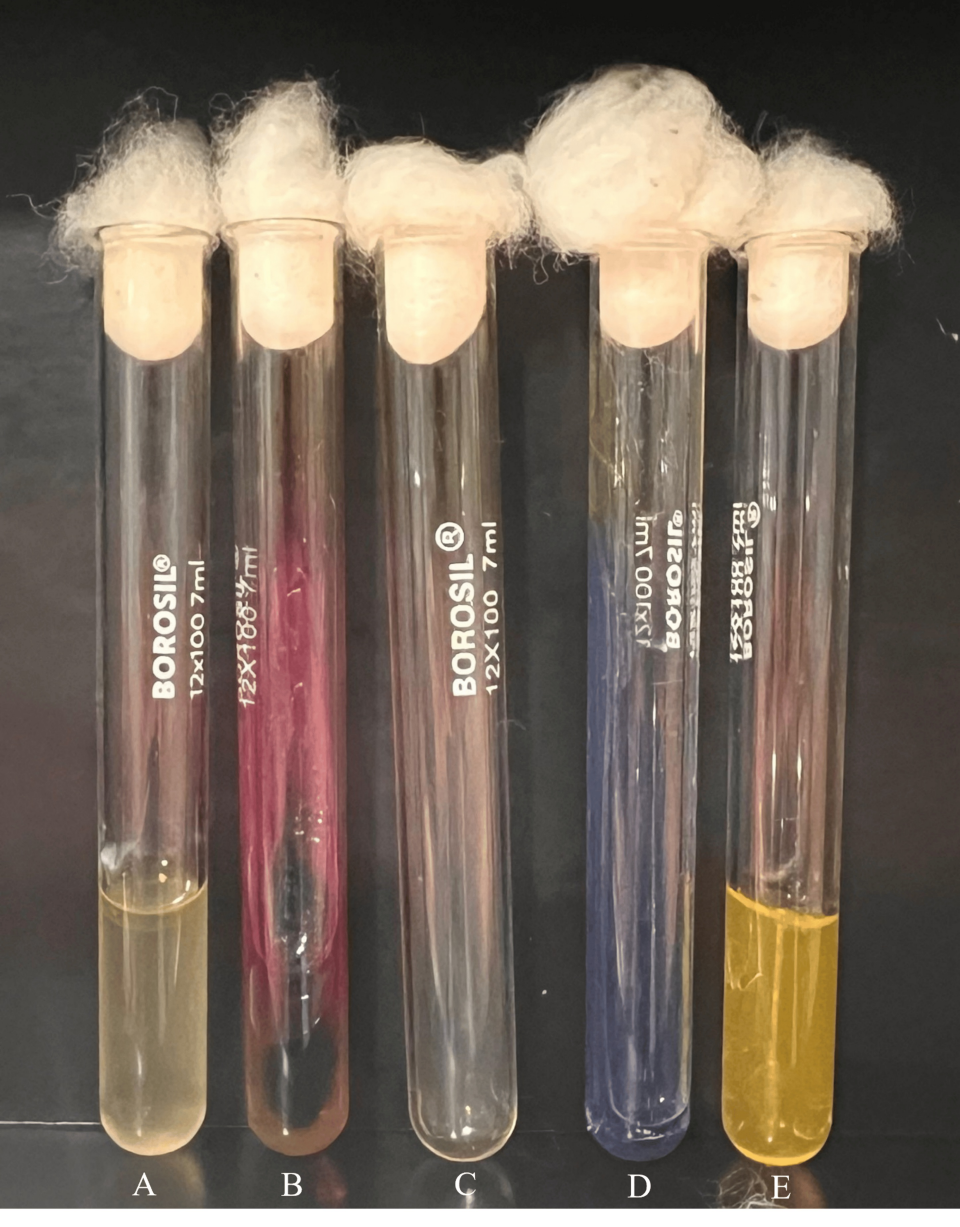
Bio-chemical identification of Salmonella Paratyphi B A: Indole: negative; B: triple sugar iron agar: alkaline slant by acidic butt, hydrogen sulfide (H2S) gas present and abundant; C: urease: not hydrolyzed; D: citrate: not utilized; E: mannitol motility medium: motile and fermented

The isolate was identified biochemically as *Salmonella *Paratyphi B. The biochemical findings were as follows: indole, negative; triple sugar iron agar, alkaline slant by acidic butt, hydrogen sulfide (H_2_S) gas present and abundant; urease, not hydrolyzed, citrate, not utilized; and it was motile and fermented in mannitol medium (Figure 5).

**Figure 6:**
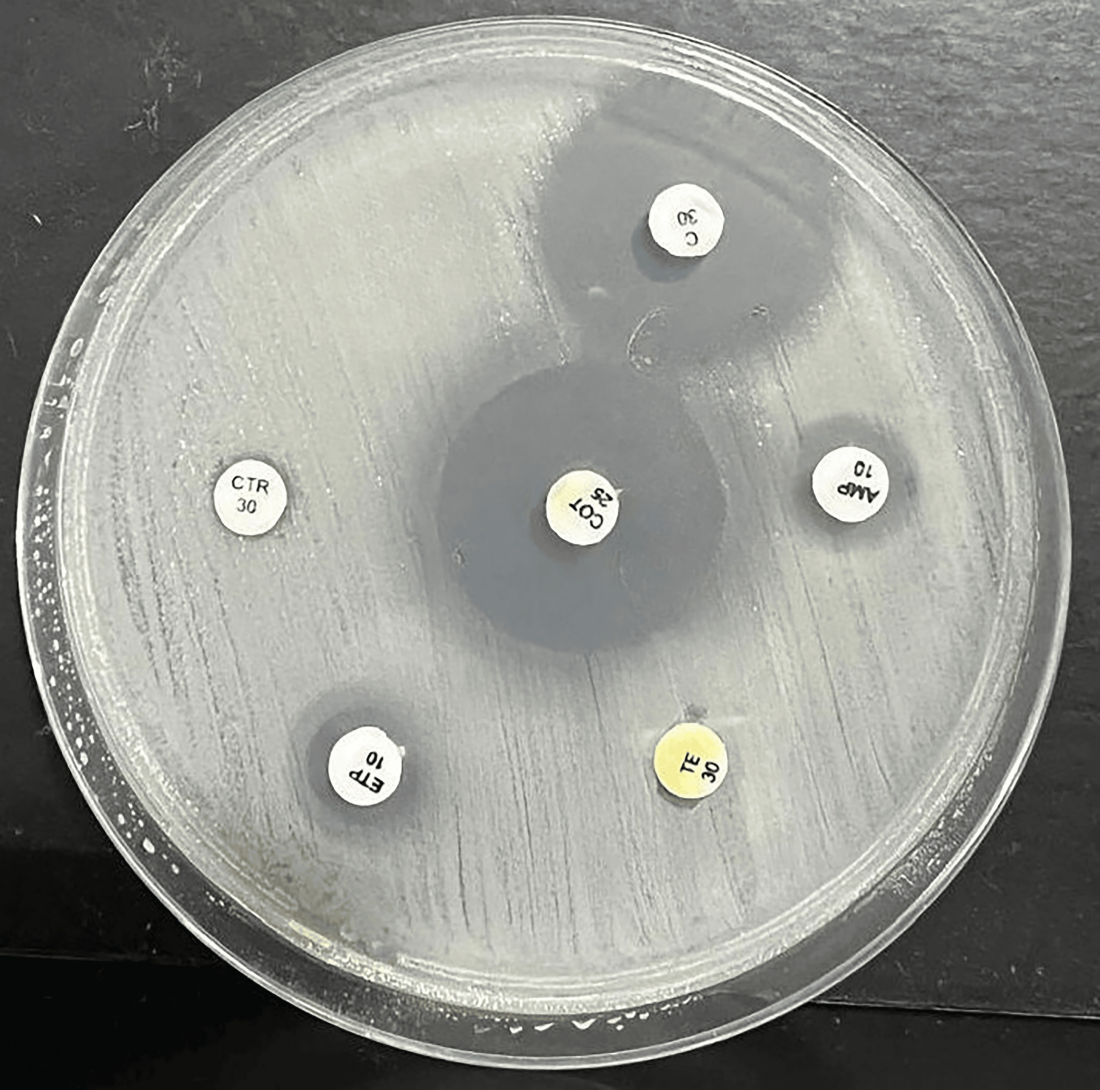
Antimicrobial susceptibility testing using the Kirby-Bauer disk diffusion method C: chloramphenicol; AMP: ampicillin; TE: tetracycline. ETP: ertapenem; CTR: ceftriaxone; COT: cotrimoxazole

Antimicrobial susceptibility testing using the Kirby-Bauer disk diffusion method showed that *Salmonella *Paratyphi B was susceptible to chloramphenicol and cotrimoxazole but resistant to all other tested antibiotics, as depicted in Figure 6.

Serotyping of the isolate was confirmed using slide agglutination and the Kauffmann-White scheme, which revealed a 2+ agglutination. Simultaneously, blood and urine samples from the patient were collected and found to be sterile with no growth. The patient underwent wound dressing and was treated with antibiotics. Based on the susceptibility profile, the antibiotic regimen was changed from meropenem to an intravenous infusion of cotrimoxazole, administered at a dose of 16 mg/80 mg over 90 minutes, twice daily. Significant improvement was noted. Upon discharge, the patient was prescribed oral cotrimoxazole at a dose of 960 mg twice daily and advised to return for a follow-up after two weeks. At the two-week follow-up, the patient's condition had markedly improved. The fever had subsided, and the foot ulcer exhibited signs of healing after two weeks. There were no indications of systemic infection, and the patient's vital signs were stable. The patient responded well to the treatment without any complications. The importance of ongoing care and regular follow-up appointments was emphasized.

## Discussion

*Salmonella *spp. are true pathogens, capable of causing both intestinal and extra-intestinal infections in humans [8]. The clinical impact of *Salmonella *spp. causing extra-intestinal manifestations is not brought to light due to the lack of documented evidence showing the actual prevalence of these infections [9]. Atypical *Salmonella *infections may cause clinical symptoms that differ from the classic gastrointestinal symptoms. The unusual manifestations include skin and soft tissue infections, urinary tract infections, and respiratory tract infections [10], but invasive infections occurring in blood, bone, joints, and meninges may be fatal, requiring appropriate management [11]. This can create a diagnostic challenge since these symptoms may resemble other conditions, leading to delays in diagnosis and appropriate treatment [12].

In India, multidrug-resistant strains of *Salmonella *have been isolated since 1960 and were first noted in Calicut [13]. Multidrug-resistant *Salmonella *Typhi refers to strains resistant to the first-line antibiotics typically used to treat typhoid fever, namely ampicillin, cotrimoxazole, and chloramphenicol [14]. A study conducted in North India observed that the prevalence of MDR *Salmonella *strains increased from 53.6% in 1997 to 63.9% in 2001. Among these resistant strains, 70% exhibited resistance to amikacin, third-generation cephalosporins, and ciprofloxacin [15]. Monitoring antimicrobial resistance patterns is crucial to guide the clinician in choosing appropriate antibiotics and avoiding unnecessary drug exposure. *Salmonella *infection remains a major epidemic, especially in developing countries. A few studies suggested carbapenems as the drug of choice for treating infections caused by strains resistant to cefotaxime and ciprofloxacin [16]. In any underdiagnosed extraintestinal infection, there is always a possibility of salmonellosis, which needs to be ruled out. Timely diagnosis and adequate management with appropriate antimicrobial therapy play a major role in reducing morbidity and mortality [17].

In our study, we observed a patient with uncontrolled diabetes affected by *Salmonella* Paratyphi B, which showed susceptibility to chloramphenicol and cotrimoxazole and resistance to other panels of antibiotics. After a change in the treatment regimen, we observed a remarkable change and clinical improvement in his condition.

## Conclusions

The patient's clinical improvement with antibiotics marked a successful outcome in a rare case of atypical typhoidal *Salmonella *wound infection. This underscores the importance of swift diagnosis and targeted treatment in effectively managing such infections with minimal risk. Healthcare providers, notably physicians and microbiologists, must remain vigilant about the potential for atypical *Salmonella *infections to ensure timely identification and appropriate therapeutic interventions. These infections, although infrequent, present diagnostic complexities due to their uncommon clinical presentations and potential resistance to standard antibiotic therapies. Practitioners should consider atypical *Salmonella *in the differential diagnosis of patients presenting with unusual symptoms of wound infections, particularly in regions where *Salmonella *prevalence or antibiotic resistance rates are elevated. This case emphasizes the critical role of tailored treatment strategies informed by antimicrobial susceptibility testing. Continued awareness of emerging resistance patterns and prompt adjustment of treatment protocols are essential for optimizing patient care and preventing the dissemination of resistant strains within healthcare settings.
